# Preoperative risk factors for postoperative complications in endoscopic pituitary surgery: a systematic review

**DOI:** 10.1007/s11102-017-0839-1

**Published:** 2017-09-15

**Authors:** Daniel J. Lobatto, Friso de Vries, Amir H. Zamanipoor Najafabadi, Alberto M. Pereira, Wilco C. Peul, Thea P. M. Vliet Vlieland, Nienke R. Biermasz, Wouter R. van Furth

**Affiliations:** 1 Department of Neurosurgery, Leiden University Medical Center, Albinusdreef 2, 2333 ZA Leiden, The Netherlands; 2 Division of Endocrinology, Department of Medicine, Leiden University Medical Center, Albinusdreef 2, 2333 ZA Leiden, The Netherlands; 3 Department of Orthopedic Surgery, Leiden University Medical Center, Albinusdreef 2, 2333 ZA Leiden, The Netherlands

**Keywords:** Pituitary, Endoscopic transsphenoidal surgery, Risk factors, CSF leak, Bleeding, Diabetes insipidus

## Abstract

**Background:**

The ability to preoperatively predict postoperative complication risks is valuable for individual counseling and (post)operative planning, e.g. to select low-risk patients eligible for short stay surgery or those with higher risks requiring special attention. These risks however, are not well established in pituitary surgery.

**Methods:**

We conducted a systematic review of associations between preoperative characteristics and postoperative complications of endoscopic transsphenoidal surgery according to the PRISMA guidelines. Risk of bias was assessed through the QUIPS tool.

**Results:**

In total 23 articles were included, containing 5491 patients (96% pituitary adenoma). There was a wide variety regarding the nature and number of risk factors, definitions, measurement and statistics employed, and overall quality of mainly retrospective studies was low. Consistent significant associations were older age for complications in general, and intraventricular extension for cerebrospinal fluid (CSF) leaks. Associations identified in some but not all studies were younger age, increased BMI, female gender, and learning curve for CSF leaks; increased tumor size for complications in general; and Rathke’s cleft cysts for diabetes insipidus. Mortality (incidence rate 1%) was not addressed as a risk factor.

**Conclusion:**

Based on current literature, of low to medium quality, it is not possible to comprehensively quantify risk factors for complications. Nevertheless, older age and intraventricular extension were associated with increased postoperative complications. Future research should aim at prospective data collection, reporting of outcomes, and uniformity of definitions. Only then a proper risk analysis can be performed for endoscopic pituitary surgery.

**Electronic supplementary material:**

The online version of this article (doi:10.1007/s11102-017-0839-1) contains supplementary material, which is available to authorized users.

## Introduction

Over the past two and a half decades, pituitary surgery has undergone major technical developments, the introduction of the endoscope perhaps being the most important one. Several systematic reviews show relatively better results in terms of gross total resection, with reduced complication rates for endoscopic surgery compared to microscopic surgery [1–10]. These complication rates in endoscopic transsphenoidal surgery (ETS) are relatively low; however, they can still be significant. In clinical decision-making, it is important to identify patients with an increased risk preoperatively for obvious reasons, e.g. planning and timing of the surgical intervention. Identified individual risk factors may be used to stimulate awareness in an attempt to reduce complication rates, improve patient counseling, and identify patients with an expected low-risk procedure, eligible for short stay surgery. Consultation with or even referral to a center of excellence is warranted in high risk patients to consider different surgical and alternative treatment strategies, such as medication and radiotherapy. A considerable number of clinical studies have reported on risk factors for complications after ETS; however, a systematic overview of the literature is lacking. The present study, therefore, aimed to systematically review the literature on preoperative risk factors for complications after ETS for pituitary tumors.

## Methods

A systematic review was conducted according to a predefined protocol, which was based on the PRISMA criteria for systematic reviews [11] and registered in Prospero, registration number CRD42017057835. The selection of studies, extraction of data, and assessment of the risk of bias were done by two independent reviewers (D.J.L. and F.V.). Disagreement was resolved through discussion and consensus. If discussion failed to lead to a consensus, a third researcher would be consulted; this did not occur, however.

### Search strategy

A literature search was conducted on May 15 2017, with the guidance of a trained clinical librarian (J.S.). The following databases were searched: PubMed, Embase, Web of Science, Cochrane, CINAHL, Academic Search Premier and ScienceDirect. Terms included were ‘pituitary adenoma’, ‘non-functioning adenoma’, ‘acromegaly’, ‘Cushing’s disease’, ‘prolactinoma’, ‘Craniopharyngioma’, ‘Rathke’s cleft cyst’, ‘complications’, ‘risk factors’ and ‘prognosis’, and derivatives or synonyms of these words. The complete search strategy can be found in online supplement 1. Reference checking of included studies was performed to screen for additional studies.

### Inclusion of articles

Inclusion criteria were: (1) articles reporting on outcomes of ETS for pituitary tumors; (2) describing an association between ≥1 preoperative characteristics and ≥1 postoperative complications; (3) published in English; (4) peer-reviewed; (5) containing original clinical data; and (6) including >10 adult patients (>18 years). Excluded studies were: (a) microscopic, endoscopic-assisted surgery, or combined microscopic and endoscopic approaches without a separate description of endoscopic results, (b) articles without a described association, and (c) articles including >10% other pathologies than pituitary adenoma.

A meta-analysis appeared to be infeasible because of heterogeneity in (the definition of) risk factors and outcomes. In addition, the number of studies assessing the same association for a complication was too small. This review focuses on complications that directly intricate the postoperative course. Perioperative CSF leaks can be managed adequately during surgery and were therefore not included. Other reviews have addressed specific complications occurring during surgery; e.g. internal carotid artery (ICA) injuries [12] or later after discharge, e.g. delayed hyponatremia [13]. Both studies, however, also included microscopic studies. The results of these studies have been used to substantiate our present conclusions.

### Selection of studies

The selection consisted of two phases: (1) title and abstract screening for potentially eligible articles, and (2) full text screening of these articles. During both phases, the same inclusion and exclusion criteria were used. During phase 1, in case of doubt, the full text paper was retrieved. Since a variety of risk factors can be investigated within the same cohort, a decision was made not to omit overlapping cohorts.

### Data extraction

Extracted study characteristics included: institution, study period, study design, number of patients, number of procedures, percentage females, tumor type, approach, length of stay, and duration of follow-up. Preoperative factors were categorized into groups (demographics, volumetric parameters, pathology, surgical factors, and endocrine parameters) and all potential associations were categorized into complications in general, neurosurgical and endocrine complications. Risk factors were considered consistent when they were reported as significant in ≥2 independent studies. Inconsistent when ≥2 positive or negative and ≥1 neutral (non-significant) associations were reported and conflicting when ≥1 positive and ≥1 negative associations were reported.

### Risk of bias

Assessment of risk of bias was done by means of the Quality in Prognostic Studies (QUIPS) tool [14]. The QUIPS tool is the standard tool used by Cochrane to review cohort studies evaluating predictive factors for diagnosis or prognosis. The results of this evaluation were put in a “summary of findings table” (Table 1). The overall risk of bias score was assessed according to that of Lazzerini [15]. A low risk of bias was given if all six domains were scored as low, or if not more than two moderate or unknown risks of bias were identified. Moderate risk of bias was given when three or less risk of bias domains were scored moderate, or unknown, in combination with no high risk of bias. Moderate was also given when one domain was scored as a high risk of bias in combination with one or less moderate or unknown risks of bias. A high risk of bias was given when two or more domains scored a high risk of bias, or four or more moderate or unknown risk of bias.

**Table 1:** Summary of findings (risk of bias)

Authors	Study participation	Study attrition	Prognostic factor measurement	Outcome measurement	Study confounding	Statistical analysis and reporting	Overall risk of bias
Ajlan 2016	Moderate	Moderate	Moderate	Moderate	High	High	**High**
Bokhari 2013	Moderate	Moderate	Moderate	Moderate	High	High	**High**
Boling 2016	Low	Moderate	Moderate	Moderate	Moderate	Low	**High**
Cavallo 2014	Moderate	Moderate	Moderate	Moderate	High	High	**High**
Cerina 2016	Moderate	Moderate	Low	Low	Low	Low	*Low*
Chabot 2015	Low	Moderate	Low	Moderate	High	Moderate	**High**
Chi 2013	Moderate	Moderate	Low	Moderate	Moderate	Moderate	**High**
Chohan 2016	Moderate	Low	Moderate	Moderate	Moderate	Moderate	**High**
Dallapiaza 2014	Moderate	High	Moderate	Moderate	High	Moderate	**High**
Dlouhy 2012	Moderate	Moderate	Low	Moderate	Low	Low	***Moderate***
Gondim 2011	Moderate	Low	Moderate	Low	High	Low	**High**
Gondim 2015	Moderate	Low	Moderate	Moderate	High	Moderate	**High**
Hofstetter 2012	Moderate	Low	Low	Low	High	Moderate	**High**
Jakimovski 2014	Moderate	Moderate	Moderate	Moderate	High	Moderate	**High**
Jang 2016	Moderate	Low	Moderate	Low	Moderate	Moderate	**High**
Karnezis 2016	Low	Moderate	Moderate	Moderate	Moderate	Low	**High**
Leach 2010	Moderate	Low	Moderate	Moderate	High	Moderate	**High**
Qureshi 2016	Moderate	Moderate	Moderate	Moderate	Moderate	Moderate	**High**
Senior 2008	Low	Moderate	Moderate	Low	High	Moderate	**High**
Sigounas 2008	Moderate	Moderate	Moderate	Low	Low	Low	***Moderate***
Thawani 2017	Moderate	Moderate	Moderate	Moderate	Moderate	Moderate	**High**
Zhan 2015	Moderate	Moderate	Moderate	Moderate	High	Moderate	**High**
Zhang 2014	Moderate	Moderate	Moderate	Moderate	Moderate	Low	**High**

## Results

### Search

**Fig. 1:**
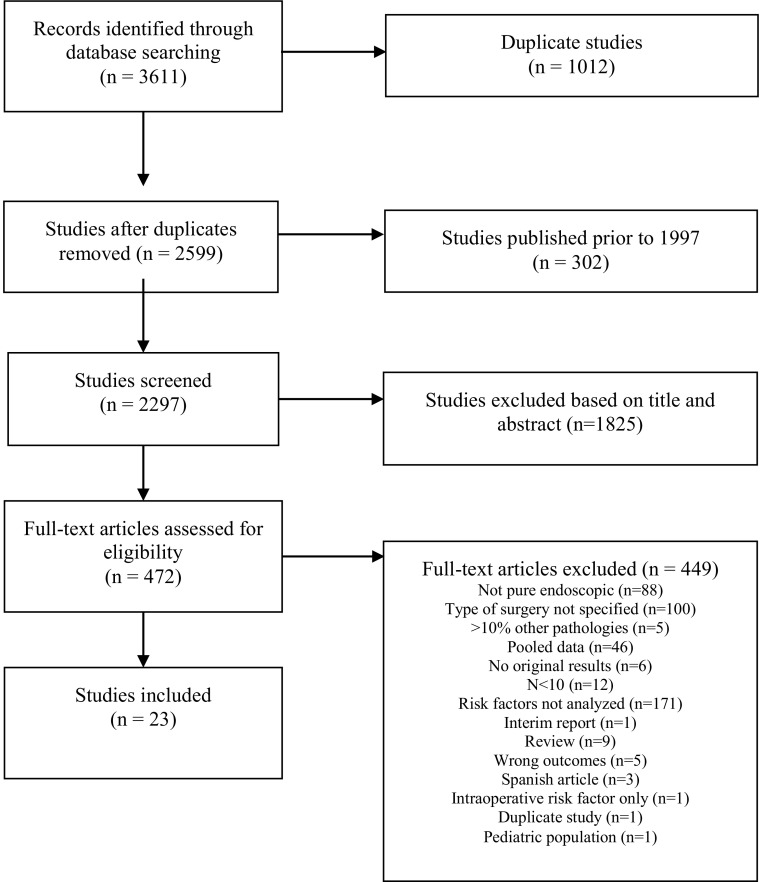
Flow chart of study assessment

The search resulted in 2596 unique titles and abstracts. The screening of titles and abstracts resulted in the selection of 472 full-text articles retrieved for the second phase of the selection process. Finally, 23 articles were included in the present systematic review (Fig. 1).

### Study characteristics

The study characteristics are summarized in Table 2. There were 3 prospective [16–18], and 20 retrospective observational cohort studies [19–27]. Publication was between 2008 and 2017 and included data from 5491 patients (median 125, IQR 78–313), of whom 2828 (52%) were female. There were 5291 pituitary adenomas (96%), 143 craniopharyngiomas (2.6%) and 39 Rathke’s cleft cysts (0.7%). Seventeen studies (61%) included patients with adenomas only [16–20, 22, 23, 27–33], five studies included a mixture of tumor types [24–26, 34, 35] and one study only included patients with craniopharyngiomas [21].

**Table 2:** Study types and demographics

Authors, institution	Study design	Study interval	Number of patients (n)	Adenomas (%)	Females (%)	Mean age (years)	Complications
Ajlan et al. [28] California, USA	Retro	2007–2012	176	100	52	50 (median)	Postoperative CSF leak 4.5%, bleeding 1.7%, epistaxis 1.1%, visual deterioration 1.1%, permanent DI 10.2%
Bokhari et al. [19] Kogarah, Australia	Retro	1998–2010	79	100	56	56.7	Postoperative CSF leak 2.5%, meningitis 0, bleeding 1.3%, visual deterioration 1.3%, transient DI 10%, permanent DI 2.5%, death 1.3%
Boling et al. [20] 6 international centers*	Retro	2002–2014	982	100	56	52	Postoperative CSF leak 5.5%, meningitis 2.7%, bleeding 3.7%, death 0.5%
Cavallo et al. [21] Naples & Bologna, Italy	Retro	1997–2012	83	0	53	50.4	Postoperative CSF leak 16.9%, meningitis 1.2%, bleeding 4.8%, visual deterioration 2.4%, overall DI 36.1%, SIADH 3.6%, death 2.4%
Cerina et al. [16] Zagreb, Croatia	Pro	2012–2013	70	100	60	44.4	Adrenal insufficiency 51.4%
Chabot et al. [22] NY (3) & Illinois, USA^†^	Retro	2009–2014	39	100	36	56.3	Postoperative CSF leak 10.3%, meningitis 2.6%, visual deterioration 0, overall DI 7.7%
Chi et al. [23] Renji, China	Retro	2011–2012	80	100	44	50.9	Postoperative CSF leak 5%, meningitis 1.3%, bleeding 0, visual deterioration 0, transient DI 11.3%, permanent DI 3.8%, death 0
Chohan et al.[29] NY, USA (1)	Retro	2003–2014	62	100	34	54.2 (median)	Postoperative CSF leak 1.6%, bleeding 1.6%, visual deterioration 9.7%, permanent DI 17.7%, death 0
Dallapiazza et al. [36] Virginia, USA	Retro	2010–2013	56	100	52	56.2	Postoperative CSF leak 7.1%, meningitis 0, epistaxis 10.7%, visual deterioration 3.6%, adrenal insufficiency 14.3%, transient DI 17.9%, permanent DI 0, SIADH 8.9%
Dlouhy et al. [24] Iowa, USA	Retro	2005–2010	92	92	52	53.0	Postoperative CSF leak 13.5%
Gondim et al.[37] Fortaleza, Brazil	Retro	1998–2009	301	100	55	42.4	Postoperative CSF leak 2.6%, meningitis 0.6%, bleeding 1.7%, epistaxis 2.0%, visual deterioration 0.3%, permanent DI 1.3%, death 1.0%
Gondim et al. [30] Fortaleza, Brazil	Retro	2000–2012	374	100	59	51	Postoperative CSF leak 3.7%, bleeding 1.6%, permanent DI 2.7%, death 1.1%
Hofstetter et al. [17] NY, USA (1)	Pro	2004–2010	71	100	46	49.9	Postoperative CSF leak 1.4%, bleeding 1.4%, visual deterioration 1.4%, permanent DI 4.2%, death 1.4%
Jakimovski et al. [18] NY, USA (1)	Pro	2003–2011	203	100	49	–	Postoperative CSF leak 3.0%, meningitis 1.0%, bleeding 1.5%, visual deterioration 1.0%, adrenal insufficiency 2.0%, overall DI 3.9%
Jang et al. [31] Changwon, South Korea	Retro	1998–2014	331	100	56	47.4	Postoperative CSF leak 1.8%, meningitis 0.6%, bleeding 0.6%, epistaxis 1.2%, transient DI 4.2%, permanent DI 0.9%, SIADH 2.7%, death 0.3%
Karnezis et al. [34] 7 international centers**	Retro	2002–2014	1161	95	54	51	Postoperative CSF leak 5.9%
Leach et al. [35] Salford, UK	Retro	2005–2007	125	87	44	51	Postoperative CSF leak 3.2%, bleeding 1.6%, epistaxis 1.6%, visual deterioration 1.6%, overall DI 4.8%, death 0
Qureshi et al. [32]Illinois, USA	Retro	2006–2012	78	100	45	52.6	Postoperative CSF leak 1.3%, transient DI 11.5%, permanent DI 2.6%
Senior et al. [25] N.C., USA	Retro	2000–2007	176	84	54	46	Postoperative CSF leak 11.4%, meningitis 1.1%, bleeding 0.5%, epistaxis 3.1%, visual deterioration 0, overall DI 20.2%, death 0.5%
Sigounas et al. [26] N.C., USA	Retro	2000–2005	110	85	52	–	Postoperative CSF leak 10.0%, transient DI 13.6%, permanent DI 2.7%
Thawani et al. [33] Pennsylvania, USA	Retro	2009–2014	203	100	49	55.7	Postoperative CSF leak 10.3%, meningitis 1.0%, bleeding 0.5%, visual deterioration 1.5%, transient DI 3.9%, permanent DI 4.4%, SIADH 5.9%, death 1.0%
Zhan et al. [27] Shandong, China	Retro	2008–2014	313	100	40	60.1	Postoperative CSF leak 3.8%, meningitis 1.6%, bleeding 0.6%, visual deterioration 1.9%, transient DI 15.4%, permanent DI 3.8%, death 0
Zhang et al. [38] Wenzhou, China	Retro	2007–2011	326	100	46	33.3	Meningitis 9.8%

(–) not assessed

(*) South Carolina, USA; Adelaide, Australia; NY, USA (2); Toronto, Canada; Atlanta, USA; Cleveland, USA

(**) South Carolina, USA; Adelaide, Australia; NY, USA (2); Toronto, Canada; Atlanta, USA; Cleveland, USA, Alabama, USA

(1) New York Presbyterian (2) Mt. Sinai Medical Center (3) Hofstra-North Shore Long Island Jewish Hospital

(†) Illinois, USA; NY, USA (3)

### Risk of bias

The results of the scoring of the methodological quality of the studies are shown in Table 1.

Overall, the methodological quality was low: only one study had a low risk of bias (4.3%), two a moderate risk (8.7%) and the remaining twenty studies had a high risk of bias (87.0%). A high risk of bias was found twelve times for study confounding (median 7.5, range 0–12).

### Complication rates

The incidence rates of complications described in the included studies are described in Table 3. The three most common complications were postoperative CSF leaks (median 4.5%, IQR 2.6–10.2%), serious bleedings (median 1.5%, IQR 0.6–1.7%), and permanent diabetes insipidus (median 2.7%, IQR 1.9–4.3%).

**Table 3:** Described risk factor per complication

	Complications in general	Postoperative CSF leak	Intracranial infection	Bleeding	Adrenal insufficiency	Transient DI	Permanent DI	Overall DI
Demographics								
Age	+^**30,31**^	=^25,33,27^, −^**20,20,24,34**^	=^27^	=^27^	=^16^	=^27^	=^27^	=^27,33^
Gender (female*)	=^31^	=^24,25,33^, +^**20,34**^			=^16^			=^25,33^
BMI		=^25^, +^**20,24,34**^						
Diabetes mellitus			+^**38**^					
Race		=^34^						=^26^
Peptic ulcer disease		+^**34**^						
Various comorbidities		=^34^						
Volumetric parameters								
Tumor size	=^22^, +^**17,31**^	=^18,25,33^, +^**25**^			+^**16**^		=^26,29,29,29^ +^**29**^	=^25,26,33^
Tumor volume	+^**17**^	=^18,24,36^					=^29^	
Intraventricular extension	+^**20**^	+^**20,21,34**^	+^**20**^	+^**20**^				
Knosp	=^22^, +^**31**^	=^36^					=^29^	
Supra-/parasellar extension	+^**31**^	+^**37**^						
Extension into the ACF	+^**20**^			+^**20**^				
Cavernous sinus invasion	=^28^	=^28,33^						
Pathology								
Tumor type	=^31^	=^18^, +^**25**^			=^16^			
Non-functioning adenoma								=^26^
Acromegaly								=^26^
Cushing’s disease		=^24^						=^26^
Prolactinoma								=^26^
RCC		+^**25**^					+^**26**^	+^**25,26**^
Craniopharyngioma		+^**34**^						=^26^
Surgical factors								
Previous radiation	+^**20**^	=^20,33^, +^**34**^		+^**20**^				
Previous surgery		=^24,33,34^					=^26,26^	=^25,26,26^
Learning curve	=^19,35^	=^19,23,32^, +^**18**^				=^32^	=^32^	=^19,23^
Endocrine parameters								
Preoperative prolactin/TSH/testosterone/cortisol					=^16^			
Preoperative T4/IGF-1/FSH/LH					+^**16**^			
Urinary-free cortisol (nmol/24 h)					+^**16**^			

Significant associations are given in bold

*Prognostic factor has a higher change of outcome

+ Positive effect; indicating a significant higher risk

− Negative effect; indicating a significant lower risk

= Neutral; relation studied, however no significant increased/decreased risk found

### Risk factors for complications

### Complications in general

Eight studies investigated the potential risk factors for complications in general [17, 19, 20, 22, 28, 30, 31, 35]. Incidence rates of complications in general were often not given. Furthermore, various definitions were used, varying from solely treating specific complications to reporting all potential complications. This included, for instance, urinary tract infections and cardiovascular complications. Some studies did not report how they defined complications in general [19, 22].

#### Demographics

Age was assessed in two studies. Both studies found an increased risk for older age (35,36). Age was defined as a categorical parameter: (1) age ≥70 versus <60 years (32.7 vs. 10%, p < 0.05) [30] and (2) age ≥50 years versus <50 OR 2.75 (95% CI 1.18–4.32, multivariate) [31]. This shows an increased risk for higher age. Female gender was investigated in one study, however no significant effect was found [31].

#### Tumor characteristics

##### Size and volume (3 studies)

Tumor size and volume were significantly associated with increased complications in general in two out of three studies. Definitions used were (1) macroadenoma versus microadenoma OR 3.98 (2.16–5.79, multivariate) [31], (2) tumor volume >10 cm^3^ versus <10 cm^3^ OR 6.3 (1.6–25.0) [17], (3) tumor diameter >3 cm versus <3 cm OR 4.8 (1.2–18.6) [17], and (4) maximum tumor diameter (no significant effect) [22].

##### Tumor extension (4 studies)

Four out of six investigated risk factors showed increased risks for complications in general. Tumor extension was defined in five different ways: (1) intraventricular extension, (2) Knosp grade, (3) supra-/parasellar extension, (4) extension into the anterior cranial fossa (ACF), and (5) cavernous sinus invasion. Intraventricular extension OR 7.85 (2.88–21.43) [20], supra-/parasellar extension (29.9 vs. 7.5%, p = 0.002) [31], and extension into the ACF OR 1.92 (1.03–3.6) [20] were significantly associated with an increased risk in one study per risk factor.

##### Tumor type (1 study)

Tumor type was investigated in one study; however, no significant effect was detected [31].

#### Surgical factors

Previous radiation was associated with an increased risk in one study (OR 8.86, 95% CI 2.05–38.28) [20]. The surgeon’s learning curve was not associated with an increased risk of complications in general in two studies [19, 35].

### CSF leak

Fourteen studies investigated the potential risk factors for postoperative CSF leaks [18–21, 23–25, 27, 28, 32–34, 36, 37]. Postoperative incidence rates of CSF leaks varied between 1.4 and 16.9%. Definitions varied between clinical evidence of CSF rhinorrhea to no definition given. Described risk factors include demographics such as age, gender, BMI and comorbidity as well as pathology, several volumetric parameters, and surgical factors.

#### Demographics

##### Age (6 studies)

Younger age was inconsistently associated with a higher risk of CSF leaks. Three studies, including two with overlapping cohorts [20, 34], found a significant association for younger age in a multivariable analysis with different definitions: (1) continuous: OR 0.93 (95% CI 0.88–0.98) [24] and OR 0.98 (0.97–1.00) [34], (2) categorical <40 versus >65 years OR 5.3 (1.17–24.11) [20], and (3) 40–64 versus >65 years OR 7.9 (1.88–33.4) [20]. Covariates included were: BMI and intraoperative CSF leaks.

##### Gender (5 studies)

In two of five studies, female gender was associated [20, 24, 25, 33, 34] with a significant increase in postoperative CSF leaks [20, 34], OR 2.4 (1.24–4.63) [20] and p = 0.045 [34]. These two large studies (n = 982; 1162) had overlapping cohorts.

##### Body mass index (BMI) (4 studies)

As expected, three out of four studies found a significant increase in postoperative CSF leaks in patients with a higher BMI [20, 24, 25, 34]. Various definitions were used: (1) continuous, multivariate: OR 1.61 (1.10–2.29) [24] and OR 1.06 (1.01–1.06) [34], and (2) categorical: <30 versus ≥30 OR 2.10 (1.14–3.86) [20]. Covariates included were age [24] and intraoperative CSF leakage [24], and craniopharyngiomas [34]. However, again, two of these studies had a large overlap in included patients [20, 34].

##### Miscellaneous (1 study)

One study evaluated various comorbidities in relation to CSF leaks (Table 3) and found a significant association only for peptic ulcer disease (p = 0.029) [34].

#### Tumor characteristics

##### Tumor size or volume (5 studies)

Only one out of five studies looking at tumor size or volume found a significant association with CSF leaks [18, 24, 25, 33, 36]. These five studies, used various cut-off values ranging from below or above 10 mm to a continuous parameter of size. The only significant association was found for tumors larger than 10 mm, compared to the smaller group, suggesting that indeed microadenoma have a lower risk for CSF leaks (34 vs. 17%, p = 0.04) [25].

##### Tumor extension (7 studies)

Tumor extension was analyzed in seven studies [20, 21, 28, 33, 34, 36, 37], however, again, defined in four different ways: (1) intraventricular extension [20, 21, 34], (2) supra-/parasellar extension [37], (3) Knosp grade [36], and (4) cavernous sinus invasion [28, 33]. Intraventricular extension was investigated by three studies: (1) 8.9 versus 27.8% [21], (2) OR 9.49 (2.97–30.26) [20], and (3) OR 3.58 (1.70–7.59) [34]. Presence of supra-/parasellar extension was investigated by one study OR 8.08, p = 0.02 [37]. Knosp grade and cavernous sinus invasion were investigated by three studies; however, none of the studies found a significantly increased risk for postoperative CSF leaks [28, 33, 36]. In summary, intraventricular extension is a clear adverse factor, while supra-/parasellar extension is only confirmed in one study.

##### Pathology (4 studies)

Four studies looked at the relationship between various forms of pathology and CSF leaks [18, 24, 25, 34]. Three studies looked at associations of individual tumor types, namely for Cushing’s disease [24], craniopharyngioma [34] and RCC [25]. RCC OR 2.6 (p < 0.001) [25], craniopharyngioma versus adenoma patients (p < 0.001) [34] and Cushing’s disease, no association [24]. Inconsistent results were found for two studies looking at tumor type in general as a predictor for postoperative CSF leaks. As expected, cystic lesions, i.e. craniopharyngioma and RCC, appear to harbor the highest risks; however, they were only described once per risk factor.

#### Surgical factors

##### Previous surgery (3 studies)

Previous surgery was not reported as a risk factor for CSF leaks [24, 33, 34].

##### Radiation (3 studies)

Even though the frequency of surgical resection after radiotherapy is low, one study found an increased risk for patients with prior radiotherapy; 4/14 patients had a postoperative CSF leak [34]. The other two studies did not find an association [20, 33].

##### Learning curve (4 studies)

One out of four studies considering the surgeon’s learning curve found a significant increase in postoperative CSF leaks [18, 19, 23, 32]. Different cut off values were used in all four: (1) early (27 cases), middle (26 cases) and late (26 cases): no significant effect [19], (2) case 1–40 versus 41–80: no significant effect [23], (3) case 1–50 versus 51–203: 10 versus 0.7% (p = 0.004) [18], and (4) case 1–9 versus 10–78: no significant effect [32]. In summary, learning curve is an inconsistent risk factor, which only showed an effect after >50 cases in one study.

### Intracranial infections

Two out of three studies reporting associations found a significant association for intracranial infections [20, 27, 38]. Intracranial infections had an incidence rate of 0–9.8%. Definitions varied from no definitions to symptomatology in combination with positive CSF cultures. Assessed risk factors were: (1) age (no significant effect) [27], (2) diabetes mellitus: OR 5.47 (1.09–6.49) [38] and intraventricular extension: OR 11.91 (3.64–38.95) [20]. Included covariates for diabetes mellitus were increased duration of surgery and CSF leakage.

### Bleeding

Only two studies looked at risk factors for bleedings: (1) ICA injury [20], and (2) postoperative intracranial bleeds (hemorrhages) [20, 27]. Incidence rates for bleedings ranged between 0 and 4.8%. Risk factors for ICA injury included prior radiation OR 44.00 (3.73–519.00) and intraventricular extension OR 13.2 (1.35–128.91) [20]. Extension into the ACF OR 4.41 (2.04–9.51) was a risk factor for intracranial bleeds [20]. One study looked at age but did not find a significant association for intracranial bleeds [27].

### Diabetes insipidus (DI)

Eight studies looked at risk factors for DI [19, 23, 25–27, 29, 32, 33]; incidence rates of DI ranged between 0.9 and 36.1%. Various definitions were used: (1) transient DI, (2) permanent DI, and (3) overall DI.

#### Demographics

Age (2 studies) [27, 33], gender (2 studies) [25, 33] and race (1 study) [26] were not associated with a significant increase of DI (all three definitions).

#### Tumor characteristics

##### Tumor size or volume (4 studies)

One study reported an association with permanent or overall DI [25, 26, 29, 33]. Various definitions were used: (1) transverse length >4 cm (no percentage given, p = 0.02) [29], (2) cranio-caudal length (no significant effect) [29], (3) antero-posterior length (no significant effect) [29], (4) maximum cross-sectional length (no significant effect) [29], (5) tumor volume >10 cm^3^ (no significant effect) [29], (6) continuous (no significant effect) [25, 33], and (7) micro- versus macroadenoma (no significant effect) [26].

##### Tumor extension (1 study)

Knosp grade was not associated with a significant increase of DI [29].

##### Pathology (2 studies)

In overlapping cohorts, RCC was significantly associated with an increased risk of DI compared to other tumor types: (1) 47.6 versus 20.2% (p < 0.05) [25], and (2) 50 versus 12%, p < 0.05 [26]. Other pathologies were not associated with DI.

#### Surgical factors

##### Previous surgery (3 studies)

Previous surgery was defined as (1) prior non-endoscopic surgery [26], (2) prior endoscopic surgery [26], and (3) prior surgery [25]. None of them was associated with an increased risk.

##### Learning curve (3 studies)

Learning curve was assessed in three studies, but not associated with an increased risk of DI [19, 23, 32].

### Adrenal insufficiency

One out of three studies looking at adrenal insufficiency addressed potential associations [16]. Incidence rates ranged between 2.0 and 51.4%. Definitions varied between extensive descriptions of used tests, while others only reported an insufficiency. Significant associations were found for tumor size, preoperative T4, IGF-1, FSH, LH and urinary-free cortisol. Corrected for tumor type, patients with larger tumors had an increased risk of adrenal insufficiency (OR 1.07, 95% CI 1.01–1.13). No association was found for gender, age, tumor type, prolactin, TSH, testosterone, and cortisol.

### Other complications of interest

Three complications were only analyzed once: cranial nerve injury, vision loss and sinusitis. One study found a significant relationship between patients with a history of an extrasellar tumor and cranial nerve injury (OR 5.94, 95% CI 1.26–28.06) [20]. One study looked at older age and vision loss [27], while one study looked at learning curve and postoperative sinusitis [32]; both, however, did not find a significant association [27, 32]. Within the endoscopic literature, no risk factors for SIADH or mortality were found.

**Table 4:** Summary of preoperative risk factors for postoperative complications

	Complications in general	CSF leak	Intracranial infections	Bleedings	Adrenal insufficiency	DI
Amendable						
Learning curve	=	Decreased after >50 cases				=
Non-amendable						
Age	Increased ≥50–70 years	Increased <65 years	=	=	=	=
Gender	=	Increased in females			=	=
BMI		Higher BMI or >30 kg/m^2^				
Diabetes mellitus			Increased risk			
Race			=			=
Peptic ulcer disease		Increased risk				
Large or giant tumors	Increased in larger tumors	Increased in larger tumors	=	=	Increased in larger tumors	Increased >1 cm
Invasive tumors	Increased by increased extension	Increased by increased extension	Increased by increased extension	Increased by increased extension	=	=
Tumor type	=	Increased in RCC/craniopharyngioma			=	Increased in RCC
Previous radiation	Increased risk	Increased risk		Increased risk		
Previous surgery		=				=
Preoperative hypopituitarism/T4/IGF-1/FSH/LH/UFC					Increased risk	

Risk ratios can be found in supplementary table 2 (online supplement)

= Neutral; relation studied, however no significant increased/decreased risk found

## Discussion

This systematic review on preoperative risk factors for postoperative complications in ETS identified only two consistent risk factors: older age for complications in general and intraventricular extension for CSF leakage. Clear and uniform definitions of postoperative complications were mostly missing and almost all studies were retrospective. This resulted in a lack of standard reporting of complications, causing a large variation between studies regarding reported risk factors and incidence rates of complications.

The most frequently studied complication, CSF leaks, was consistently associated with intraventricular extension. Other risk factors were not consistent, but did not report conflicting results. At this stage, we conclude that intraventricular extension increases the risk of CSF leaks and lower age, female gender, and high BMI potentially increase the risk (Table 4). The second most studied association was complications in general, for which we conclude that patients with older age (cut off ≥50–70 years) have an increased risk of complications in general. Although tumor size, volume and extension showed inconsistent results, results indicate an increased risk for larger tumors and tumors with an invasive growth pattern. The third most studied complication was DI. Although found in overlapping cohorts, a significant association was found between RCC and DI. Results reported between DI and tumor size were inconsistent but were not conflicting. Therefore, increased tumor size/volume might increase the risk of DI. This should, however, be further investigated. For several other complications, i.e. intracranial infections, serious bleedings and adrenal insufficiency, described associations have only been reported once. Further confirmation of whether these risk factors indeed increase the risk of complications is needed.

A distinction can be made between amendable and non-amendable risk factors. Even though often difficult to change, these should be taken into consideration in cases with increased risks. The learning curve is perhaps one of the easiest to amend. Experience (learning curve), for instance, appears to be an important risk factor for a lower risk of CSF leaks. This was confirmed in some but not all studies, while learning curve was not associated with any other complication. Obviously, learning curve statistics can be biased since a more experienced surgeon will operate on a more complex case mix with an innate higher risk of CSF leaks, which cannot be extracted from the currently available series. In a national survey, Ciric found that for most complications, surgical learning curve is an important factor [39]. This was assessed in 1997, however, when ETS was not commonplace. Based on the available literature, we advise taking the learning curve into account and consider referral to a center of excellence in cases that harbor an increased risk for CSF leak in itself, e.g. patients with intraventricular extension, lower age, females and in patients with obesity.

Increased BMI and younger age were risk factors for postoperative CSF leaks. This might be explained by the increased intra-abdominal pressure [40]. Perhaps when time permits, one should motivate patients to lose weight to reduce risks of CSF leaks postoperatively. While age increases in the course of time, older age is also a risk factor for complications in general. One should therefore weigh the risks.

Since generally only large tumors have suprasellar, intraventricular extension or extension into the ACF, these risk factors can be considered correlated and classified under tumor size. When taking this into account, large or giant pituitary tumors are associated with complications in general and postoperative CSF leaks. Literature on endoscopic resection of giant adenoma is scarce, however increased risk of complications can be found [41]. In particular, intratumoral bleeding rates or postoperative apoplexy in tumor residual have been reported, ranging from 2.1 to 3.7% [6, 42, 43]. One could argue that in firm tumors, a combined endoscopic transsphenoidal and open transcranial approach is safest for giant adenomas to maximize tumor resection [43–45]. Even though tumor size is often not amendable, in select cases one might consider medication to decrease tumor size, making it manageable for surgery and subsequently potentially lower the risk of complications, although this strategy is not evidence-based. However, the downside of medication is that it could change the tumor characteristics, making resection less manageable. Whether tumor shrinkage due to medication improves complication risks is not assessed.

Pathology, also a non-amendable risk factor, might also be an important risk factor for postoperative complications. In particular, several associations were described for RCCs and craniopharyngiomas; however, only those for DI have been reported more than once (in overlapping cohorts). These two tumors have an increased risk of DI, possibly also for postoperative CSF leaks. The relationship with pathology can likely be explained by the tumor etiology. Whereas RCCs are typically located between the anterior and posterior lobe, compression/manipulation of the posterior lobe is likely to occur prior to or during surgery. Craniopharyngiomas commonly arise in the pituitary stalk, which is vulnerable to surgical manipulation; therefore, surgery is more likely to cause DI. Another risk factor found for RCCs was postoperative CSF leaks.

Despite being addressed only in one study, previous radiation showed an association between complications in general and carotid artery injury. Even though radiotherapy prior to surgery is not common, some adenomas are very therapy resistant and need additional surgery. Boling et al. presented data from nine patients who had received radiotherapy prior to surgery, showing a complication rate of 33% [20]. This might be explained by induced fibrosis, atrophy and vascular damage [46], changing the tissue structure and characteristics, making it more fragile. While presenting results of microscopic surgery, Laws also described an increased risk of vascular injury due to radiation therapy [47].

### Comparison with other systematic reviews

One of the most reviewed subjects in pituitary surgery literature is the comparison between endoscopic and microscopic surgery. We found several reviews assessing the topic. Because the influence of surgical technique was the primary interest of comparison, patient-related risk factors were not investigated in these reviews. Typically, gross-total resection and complications have been compared between microscopy and endoscopy, most showing equal or superior results in favor of endoscopy; however, patient-related risk factors have not been further determined [1–10]. In the undivided (microscopic and endoscopic combined) literature, the following preoperative risk factors were found for delayed symptomatic hyponatremia by Cote [13]: higher age, female gender, larger tumor size, and Cushing disease. In the present review of endoscopic literature, no associations for delayed hyponatremia, or SIADH, and preoperative risk factors were found, indicating that these are either not relevant for endoscopic resections, or not yet adequately studied.

## Limitations and future perspectives

The main purpose of the study was to improve preoperative patient counseling and to identify high- and low-risk patients. The low quality of the studies precludes firm conclusions based on this review. Overall, most studies were retrospective and too small to allow multivariable analyses. Also, no meta-analysis could be performed because of the heterogeneity and low number of associations. Complications were often defined differently and mostly gave limited descriptions. This complicates generalizability, and future researchers should aim at clearly defining (presented) complications in an effort to improve the clinical impact of future research on daily practice. Furthermore, reporting of outcomes, not only by centers of excellence, and prospective registration will lead to further evolvement in the field. Many examples, like the Value Based Healthcare concept [48], have shown that improvement in reporting of outcomes and better registration lead to improvements in patient-relevant outcomes.

We realize that only a subset of the total number of studies reporting on complication rates in ETS could be included in this study since the vast majority did not perform risk factor analysis. Furthermore, studies that presented only pooled data between microscopic and endoscopic surgery did not give a utilizable overview of potential risk factors for complications specific for patients treated through an endoscopic transsphenoidal approach, as in many daily practices nowadays.

Although many studies assessed individual risk factors of different types of postoperative complications, there were no prognostic models found in the current literature. Prognostic models in other fields have shown added value in individualized decision-making and patient counseling. Such a model could have different types of outcomes, based on the aim of the model: complications in general, prediction of potential candidates for short stay. Before implementation of such a model, it should be thoroughly internally and externally validated.

Although on average the reported mortality rate is around 0.6%, unfortunately no associations were found in the current literature. Even though incidence rates are low, they are not negligible. Suggested improvements for definitions and registration of complications might give us a better understanding of the etiology of these complications.

## Conclusion

We present an overview of preoperative risk factors for postoperative complications. Only two risk factors were consistently associated with increased risks: older age for complications in general and intraventricular extension for CSF leakage. This does not mean that there are no other important risk factors, and further emphasizes the need for uniform definitions, reporting of outcomes and prospective registration. The low methodological quality of included studies, inconsistent results, and lack of uniform definitions make firm conclusions difficult. Nevertheless, we believe that awareness of presented risks may benefit patient counseling and surgical case selection.

## Electronic supplementary material

Below is the link to the electronic supplementary material.

Supplementary material 1 (DOCX 15 KB)

Supplementary material 2 (DOCX 19 KB)

Supplementary material 3 (DOCX 33 KB)

## Abbreviations

ACF: Anterior cranial fossa
BMI: Body mass index
CSF: Cerebrospinal fluid
DI: Diabetes insipidus
ETS: Endoscopic transsphenoidal surgery
ICA: Internal carotid artery
QUIPS: Quality in Prognostic Studies
RCC: Rathke’s cleft cyst
SIADH: Syndrome of inappropriate anti-diuretic hormone (secretion)

## References

[1] Bastos RVS, Silva CM, Tagliarini JV, Zanini MA, Romero FR, Boguszewski CL (2016). Endoscopic versus microscopic transsphenoidal surgery in the treatment of pituitary tumors: systematic review and meta-analysis of randomized and non-randomized controlled trials. Arch Endocrinol Metab.

[2] Ammirati M, Wei L, Ciric I (2013). Short-term outcome of endoscopic versus microscopic pituitary adenoma surgery: a systematic review and meta-analysis. J Neurol Neurosurg Psychiatry.

[3] Deklotz TR, Chia SH, Lu W, Makambi KH, Aulisi E, Deeb Z (2012). Meta-analysis of endoscopic versus sublabial pituitary surgery. Laryngoscope.

[4] Gao Y, Zhong C, Wang Y, Xu S, Guo Y, Dai C (2014). Endoscopic versus microscopic transsphenoidal pituitary adenoma surgery: a meta-analysis. World J Surg Oncol.

[5] Goudakos JK, Markou KD, Georgalas C (2011). Endoscopic versus microscopic trans-sphenoidal pituitary surgery: a systematic review and meta-analysis. Clin Otolaryngol.

[6] Komotar RJ, Starke RM, Raper DM, Anand VK, Schwartz TH (2012). Endoscopic endonasal compared with microscopic transsphenoidal and open transcranial resection of giant pituitary adenomas. Pituitary.

[7] Li A, Liu W, Cao P, Zheng Y, Bu Z, Zhou T (2017). Endoscopic versus microscopic transsphenoidal surgery in the treatment of pituitary adenoma: a systematic review and meta-analysis. World Neurosurg.

[8] Rotenberg B, Tam S, Ryu WH, Duggal N (2010). Microscopic versus endoscopic pituitary surgery: a systematic review. Laryngoscope.

[9] Strychowsky J, Nayan S, Reddy K, Farrokhyar F, Sommer D (2011). Purely endoscopic transsphenoidal surgery versus traditional microsurgery for resection of pituitary adenomas: systematic review. J Otolaryngol Head Neck Surg.

[10] Tabaee A, Anand VK, Barrón Y, Hiltzik DH, Brown SM, Kacker A (2009). Endoscopic pituitary surgery: a systematic review and meta-analysis. J Neurosurg.

[11] Moher D, Liberati A, Tetzlaff J, Altman DG, The PRISMA Group (2009). Preferred reporting items for systematic reviews and meta-analyses: the PRISMA statement. Ann Intern Med.

[12] Chin OY, Ghosh R, Fang CH, Baredes S, Liu JK, Eloy JA (2016). Internal carotid artery injury in endoscopic endonasal surgery: a systematic review. Laryngoscope.

[13] Cote DJ, Alzarea A, Acosta MA, Hulou MM, Huang KT, Almutairi H (2016). Predictors and rates of delayed symptomatic hyponatremia after transsphenoidal surgery: a systematic review. World Neurosurg.

[14] Hayden JA, van der Windt DA, Cartwright JL, Côté P, Bombardier C (2013). Assessing bias in studies of prognostic factors. Ann Intern Med.

[15] Lazzerini M, Sonego M, Pellegrin MC (2015). Hypoxaemia as a mortality risk factor in acute lower respiratory infections in children in low and middle-income countries: systematic review and meta-analysis. PLoS ONE.

[16] Cerina V, Kruljac I, Radosevic JM, Kirigin LS, Stipic D, Pecina HI (2016). Diagnostic accuracy of perioperative measurement of basal anterior pituitary and target gland hormones in predicting. Medicine.

[17] Hofstetter CP, Nanaszko MJ, Mubita LL, Tsiouris J, Anand VK, Schwartz TH (2012). Volumetric classification of pituitary macroadenomas predicts outcome and morbidity following endoscopic endonasal transsphenoidal surgery. Pituitary.

[18] Jakimovski D, Bonci G, Attia M, Shao H, Hofstetter C, Tsiouris AJ (2014). Incidence and significance of intraoperative cerebrospinal fluid leak in endoscopic pituitary surgery using intrathecal fluorescein. World Neurosurg.

[19] Bokhari AR, Davies MA, Diamond T (2013). Endoscopic transsphenoidal pituitary surgery: a single surgeon experience and the learning curve. Br J Neurosurg.

[20] Boling CC, Karnezis TT, Baker AB, Lawrence LA, Soler ZM, Vandergrift WA (2016). Multi-institutional study of risk factors for perioperative morbidity following transnasal endoscopic pituitary adenoma surgery. Int Forum Allergy Rhinol.

[21] Cavallo LM, Frank G, Cappabianca P, Solari D, Mazzatenta D, Villa A (2014). The endoscopic endonasal approach for the management of craniopharyngiomas: a series of 103 patients. J Neurosurg.

[22] Chabot JD, Chakraborty S, Imbarrato G, Dehdashti AR (2015). Evaluation of outcomes after endoscopic endonasal surgery for large and giant pituitary macroadenoma: a retrospective review of 39 consecutive patients. World Neurosurg.

[23] Chi F, Wang Y, Lin Y, Ge J, Qiu Y, Guo L (2013). A learning curve of endoscopic transsphenoidal surgery for pituitary adenoma. J Craniofac Surg.

[24] Dlouhy BJ, Madhavan K, Clinger JD, Reddy A, Dawson JD, O’Brien EK (2012). Elevated body mass index and risk of postoperative CSF leak following transsphenoidal surgery. J Neurosurg.

[25] Senior BA, Ebert CS, Bednarski KK, Bassim MK, Younes M, Sigounas D (2008). Minimally invasive pituitary surgery. Laryngoscope.

[26] Sigounas DG, Sharpless JL, Cheng DM, Johnson TG, Senior BA, Ewend MG (2008). Predictors and incidence of central diabetes insipidus after endoscopic pituitary surgery. Neurosurgery.

[27] Zhan R, Ma Z, Wang D, Li X (2015). Pure endoscopic endonasal transsphenoidal approach for nonfunctioning pituitary adenomas in the elderly: surgical outcomes and complications in 158 patients. World Neurosurg.

[28] Ajlan A, Achrol AS, Albakr A, Feroze AH, Westbroek EM, Hwang P (2017). Cavernous sinus involvement by pituitary adenomas: clinical implications and outcomes of endoscopic endonasal resection. J Neurol Surg B.

[29] Chohan MO, Levin AM, Singh R, Zhou Z, Green CL, Kazam JJ (2016). Three-dimensional volumetric measurements in defining endoscope-guided giant adenoma surgery outcomes. Pituitary.

[30] Gondim JA, Almeida JP, De Albuquerque LA, Gomes E, Schops M, Mota JI (2015). Endoscopic endonasal transsphenoidal surgery in elderly patients with pituitary adenomas. J Neurosurg.

[31] Jang JH, Kim KH, Lee YM, Kim JS, Kim YZ (2016). Surgical results of pure endoscopic endonasal transsphenoidal surgery for 331 pituitary adenomas: a 15-year experience from a single institution. World Neurosurg.

[32] Qureshi T, Chaus F, Fogg L, Dasgupta M, Straus D, Byrne RW (2016). Learning curve for the transsphenoidal endoscopic endonasal approach to pituitary tumors. Br J Neurosurg.

[33] Thawani JP, Ramayya AG, Pisapia JM, Abdullah KG, Lee JY, Grady MS (2017). Operative strategies to minimize complications following resection of pituitary macroadenomas. J Neurol Surg B.

[34] Karnezis TT, Baker AB, Soler ZM, Wise SK, Rereddy SK, Patel ZM, Oyesiku NM, DelGaudio JM, Hadjipanayis CG, Woodworth BA, Riley KO, Lee J, Cusimano MD, Govindaraj S, Psaltis A (2016). Factors impacting cerebrospinal fluid leak rates in endoscopic sellar surgery. Int Forum Allergy Rhinol.

[35] Leach P, Abou-Zeid AH, Kearney T, Davis J, Trainer PJ, Gnanalingham KK (2010). Endoscopic transsphenoidal pituitary surgery: evidence of an operative learning curve. Neurosurgery.

[36] Dallapiazza R, Bond AE, Grober Y, Louis RG, Payne SC, Oldfield EH (2014). Retrospective analysis of a concurrent series of microscopic versus endoscopic transsphenoidal surgeries for Knosp Grades 0–2 nonfunctioning pituitary macroadenomas at a single institution. J Neurosurg.

[37] Gondim JA, Almeida JPC, Albuquerque LAF, Schops M, Gomes E, Ferraz T (2011). Endoscopic endonasal approach for pituitary adenoma: surgical complications in 301 patients. Pituitary.

[38] Zhang L, Chen M (2014). Analysis of factors causing intracranial infection after endoscopic resection of pituitary tumors by transnasal-sphenoidal approach. Biomed Res.

[39] Ciric I, Ragin A, Baumgartner C, Pierce D (1997). Complications of transsphenoidal surgery: results of a national survey, review of the literature, and personal experience. Neurosurgery.

[40] Chai NC, Scher AI, Moghekar A, Bond DS, Peterlin BL (2014). Obesity and headache: part I—a systematic review of the epidemiology of obesity and headache. Headache.

[41] Halvorsen H, Ramm-Pettersen J, Josefsen R, Rønning P, Reinlie S, Meling T (2014). Surgical complications after transsphenoidal microscopic and endoscopic surgery for pituitary adenoma: a consecutive series of 506 procedures. Acta Neurochir.

[42] De Paiva Neto MA, Vandergrift A, Fatemi N, Gorgulho AA, Desalles AA, Cohan P (2010). Endonasal transsphenoidal surgery and multimodality treatment for giant pituitary adenomas. Clin Endocrinol.

[43] Yano S, Hide T, Shinojima N (2017). Efficacy and complications of endoscopic skull base surgery for giant pituitary adenomas. World Neurosurg.

[44] Van Lindert EJ, Grotenhuis JA (2009). The combined supraorbital keyhole-endoscopic endonasal transsphenoidal approach to sellar, perisellar and frontal skull base tumors: surgical technique. Minim Invasive Neurosurg.

[45] Nishioka H, Hara T, Usui M, Fukuhara N, Yamada S (2012). Simultaneous combined supra-infrasellar approach for giant/large multilobulated pituitary adenomas. World Neurosurg.

[46] Barnett GC, West CM, Dunning AM, Elliott RM, Coles CE, Pharoah PDP (2009). Normal tissue reactions to radiotherapy: towards tailoring treatment dose by genotype. Nat Rev Cancer.

[47] Laws ER (1999). Vascular complications of transsphenoidal surgery. Pituitary.

[48] Porter ME (2010). What is value in health care?. N Engl J Med.

