# Supplementary material for: Preoperative risk factors for postoperative complications in endoscopic pituitary surgery: a systematic review
Source: Pituitary. 2017 Sep 15;21(1):84–97. doi: 10.1007/s11102-017-0839-1 (PMC5767215; doi:10.1007/s11102-017-0839-1)
Supplement: Supplementary file 1 — Supplementary material 1 (DOCX 15 KB) [file 11102_2017_839_MOESM1_ESM.docx]

**Search strategy**

(("Pituitary Neoplasms/surgery"[majr] OR "Pituitary Diseases/surgery"[majr] OR "Pituitary ACTH Hypersecretion/surgery"[majr] OR "Pituitary Gland/surgery"[majr] OR "Craniopharyngioma/surgery"[Majr] OR (("Pituitary Neoplasms"[majr] OR "Pituitary Diseases"[majr] OR "Pituitary ACTH Hypersecretion"[majr] OR "Pituitary Gland"[majr] OR "Craniopharyngioma"[majr]) AND ("surgery"[Subheading] OR "surgery"[tw] OR surgical*[tw] OR neurosurg*[tw] OR "operation"[tw] OR "surgical procedures, operative"[mesh])) OR ((Pituitary Neoplasm*[ti] OR Pituitary Tumor*[ti] OR Pituitary Tumour*[ti] OR Pituitary Cancer*[ti] OR Pituitary Carcinoma*[ti] OR "Cushing syndrome"[ti] OR Microadenoma*[ti] OR Micro-adenoma*[ti] OR Macroadenoma*[ti] OR Macro-adenoma*[ti] OR "rathke cleft cyst"[ti] OR "rathke cleft cysts"[ti] OR "rathke cyst"[ti] OR "rathke cysts"[ti] OR "rathke s cleft cyst"[ti] OR "rathke s cleft cysts"[ti] OR "rathke s cyst"[ti] OR "rathke s cysts"[ti] OR "rathke's cleft cyst"[ti] OR "rathke's cleft cysts"[ti] OR Prolactinoma*[ti] OR Growth-hormone secreting pituitary adenoma*[ti] OR ACTH-secreting pituitary adenoma*[ti] OR Non-functioning adenoma*[ti] OR Craniopharyngioma*[ti]) AND ("surgery"[Subheading] OR "surgery"[tw] OR surgical*[tw] OR neurosurg*[tw] OR "operation"[tw] OR "surgical procedures, operative"[mesh]))) AND ("complications"[Subheading] OR "Postoperative Complications"[Mesh] OR "Intraoperative Complications"[Mesh] OR "complication"[tw] OR "complications"[tw] OR "Diabetes Insipidus"[Mesh] OR "diabetes insipidus"[tw] OR "Meningitis"[Mesh] OR "meningitis"[tw] OR "Hypopituitarism"[Mesh] OR "hypopituitarism"[tw] OR "hyponatremia"[mesh] OR "hyponatremia"[tw] OR "Visual acuity"[mesh] OR "decreased visual acuity"[tw] OR "Vision, Low"[Mesh] OR "loss of vision"[tw] OR "Hemianopsia"[Mesh] OR "hemianopsia"[tw] OR "Hemorrhage"[Mesh] OR haemorrhag*[tw] OR hemorrhag*[tw] OR hematoma*[tw] OR haematoma*[tw] OR "Sinusitis"[Mesh] OR "sinusitis"[tw] OR "Hyponatremia"[Mesh] OR "hyponatremia"[tw] OR "hyponatraemia"[tw] OR "Cerebrospinal Fluid Leak"[Mesh] OR "cerebrospinal fluid leak"[tw] OR "CSF leak"[tw] OR rhinorrhea*[tw] OR "Carotid Artery Injuries"[Mesh] OR "Carotid Artery Injuries"[tw] OR "Carotid Artery Injury"[tw] OR "carotid injury"[tw] OR "carotid injuries"[tw] OR "Epistaxis"[Mesh] OR "epistaxis"[tw] OR "Pneumocephalus"[Mesh] OR "pneumocephalus"[tw] OR "Thrombosis"[Mesh] OR "thrombosis"[tw] OR "DVT"[tw] OR "Pulmonary Embolism"[Mesh] OR "pulmonary embolism"[tw] OR "Blood Transfusion"[Mesh] OR "transfusion"[tw] OR "Pneumonia"[Mesh] OR "pneumonia"[tw] OR "Respiratory Insufficiency"[Mesh] OR "Respiratory Insufficiency"[tw] OR "respiratory failure"[tw] OR "Infection"[Mesh] OR infection*[tw] OR "Heart Arrest"[Mesh] OR "Heart Arrest"[tw] OR "cardiac arrest"[tw] OR "Myocardial Infarction"[Mesh] OR " myocardial infarction"[tw] OR "Stroke"[Mesh] OR "stroke"[tw] OR "Death"[Mesh] OR "death"[tw] **OR "outcome"[tw] OR "outcomes"[tw]**) AND ("Predictive Value of Tests"[Mesh] OR "predictor"[tw] OR "predictors"[tw] OR "prediction"[tw] OR "Predictive factors"[tw] OR "Predictive factor"[tw] OR "Prediction factors"[tw] OR "Prediction factor"[tw] OR "Predictive Model"[tw] OR "Predictive Models"[tw] OR "Prediction Model"[tw] OR "Prediction Models"[tw] OR predict*[tw] OR "Prognosis"[Mesh] OR "Prognosis"[tw] OR "prognostic factor"[tw] OR "prognostic factors"[tw] OR prognostic*[tw] OR "Risk Assessment"[Mesh] OR "Risk"[mesh] OR "risk"[tw] OR "risks"[tw])) AND (english[la] OR dutch[la]) NOT ("Case Reports"[ptyp] NOT "Clinical Study"[Publication Type]) NOT ("Animals"[mesh] NOT "Humans"[mesh]) NOT ("Child"[mesh] NOT ("Adolescent"[mesh] OR "Adult"[mesh])) NOT (microscop*[ti] NOT (endoscop*[ti] OR "Endoscopy"[majr]))
