# Supplementary material for: Preoperative risk factors for postoperative complications in endoscopic pituitary surgery: a systematic review
Source: Pituitary. 2017 Sep 15;21(1):84–97. doi: 10.1007/s11102-017-0839-1 (PMC5767215; doi:10.1007/s11102-017-0839-1)
Supplement: Supplementary file 2 — Supplementary material 2 (DOCX 19 KB) [file 11102_2017_839_MOESM2_ESM.docx]

| **Supplementary table 1** Incidence of pathology | | | | | | | | | | | | |
| --- | --- | --- | --- | --- | --- | --- | --- | --- | --- | --- | --- | --- |
| **Authors** | **N** | **NFA** | **Acromegaly** | **Cushing** | **Prolactinoma** | | **TSH** | **FSH-LH** | **Craniopharyngioma** | **RCC** | **Other** | **Macro \| Giant (%)** |
| Ajlan 2016 | 176 | 106 | 24 | 17 | 27 | 2 | | 0 | 0 | 0 | 0 | 77 \| - |
| Bokhari 2013 | 79 | 39 | 19 | 4 | 16 | | 1 | 0 | 0 | 0 | 0 | 91 \| - |
| Boling 2016 | 982 | 982 adenoma, type not specified | | | | | | | 0 | 0 | 0 | - \| - |
| Cavallo 2014. | 83 | 0 | 0 | 0 | 0 | | 0 | 0 | 83 | 0 | 0 | - \| - |
| Cerina 2016 | 70 | 37 | 5 | 0 | 28 | 0 | | 0 | 0 | 0 | 0 | - \| - |
| Chabot 2015 | 39 | 39 adenoma, type not specified | | | | | | | 0 | 0 | 0 | 85 \| 15 |
| Chi 2013 | 80 | 34 | 9 | 3 | 26 | | 3 |  | 0 | 0 | 5^a^ | 80 \| - |
| Chohan 2016 | 62 | 62 adenoma, type not specified | | | | | | | 0 | 0 | 0 | - \| 100 |
| Dallapiaza 2014. | 56 | 56 | 0 | 0 | 0 | | 0 | 0 | 0 | 0 | 0 | 100 \| - |
| Dlouhy 2012 | 92 | 88 adenoma of which 5 Cushing, other types not specified | | | | | | | 0 | 3 | 5^b^ | - \| - |
| Gondim 2011 | 301 | 135 | 68 | 37 | 48 | | 1 | 12 | 0 | 0 | 0 | 66 \| 16 |
| Gondim 2015 | 374 | 374 | 0 | 0 | 0 | | 0 | 0 | 0 | 0 | 0 | 100 \| - |
| Hofstetter 2012. | 71 | 45 | 14 | 2 | 10 | | 0 | 0 | 0 | 0 | 0 | 72 \| 28 |
| Jakimovski 2014 | 203 | 138 | 28 | 14 | 23 | | 0 | 0 | 0 | 0 | 0 | - \| - |
| Jang 2016 | 331 | 157 | 20 | 29 | 104 | | 2 | 6 | 0 | 0 | 13^c^ | 70 \| - |
| Karnezis 2016 | 1161 | 1108 adenoma, type not specified | | | | | | | 53 | 0 | 0 | - \| - |
| Leach 2010 | 125 | 67 | 22 | 10 | 9 | | 1 | 0 | 4 | 3 | 9^d^ | 85 \| - |
| Qureshi 2016 | 78 | 78 adenoma, type not specified | | | | | | | 0 | 0 | 0 | 96 \| - |
| Senior 2008 | 176 | 94 | 15 | 20 | 10 | | 0 | 0 | 2 | 21 | 15^e^ | 77 \| - |
| Sigounas 2008 | 110 | 61 | 15 | 9 | 9 | | 0 | 0 | 1 | 12 | 3^f^ | 71 \| - |
| Thawani 2017. | 203 | 74 | 21 | 19 | 7 | 1 | | 32 | 0 | 0 | 0 | 100 \| - |
| Zhan 2015 | 313 | 313 | 0 | 0 | 0 | | 0 | 0 | 0 | 0 | 0 | 82 \| - |
| Zhang 2014 | 326 | 70 | 45 | 36 | 175 | | 0 | 0 | 0 | 0 | 0 | 76 \| 14 |
| - not assessed  ^a^ 5 mixed  ^b^ 4 connective tissue/infection, 1 metastatic lesion  ^c^ 4 PRL-ACTH-secreting adenomas, 9 PRL-GH-secreting adenomas  ^d^ 6 apoplexy, 1 pituicytoma, 1 pituitary dermoid, 1 clival chordoma  ^e^ 9 mixed, 2 chordomas, 1 FAS-secreting (Fatty Acid Synthetase), 2 metastasis, 1 lymphocytic hypophysitis  ^f^ 2 chordoma, 1 FAS-secreting | | | | | | | | | | | | |
