# Supplementary material for: Preoperative risk factors for postoperative complications in endoscopic pituitary surgery: a systematic review
Source: Pituitary. 2017 Sep 15;21(1):84–97. doi: 10.1007/s11102-017-0839-1 (PMC5767215; doi:10.1007/s11102-017-0839-1)
Supplement: Supplementary file 3 — Supplementary material 3 (DOCX 33 KB) [file 11102_2017_839_MOESM3_ESM.docx]

| **Supplementary table 2** Summary of study characteristics of prognostic cohort studies | | | | | |
| --- | --- | --- | --- | --- | --- |
| **First author, year** | **Study Quality** | **Study population** | **Prognostic factor** | **Outcome** | **Association** |
| Ajlan et al., 2016 | High Risk | Patients with pituitary adenomas extending in the cavernous sinus (CS) vs. no CS invasion, N=176 | CS involvement | Complications in general | = |
|  |  |  |  | Postoperative CSF leak | = |
| Bokhari et al.,  2013 | High Risk | Consecutively treated adenomas, N=79 | Learning curve  (cut off 27/26/26) | Complications in general | = |
|  |  |  |  | Postoperative CSF leak | = |
|  |  |  |  | Overall DI | = |
| Boling et al., 2016 | High Risk | Multicenter cohort study in pituitary adenomas, N=982 | Previous radiation | Complications in general | OR 8.86, 95% CI 2.05-38.28, p=0.003 |
|  |  |  | Intraventricular extension |  | OR 7.85, 95% CI 2.88-21.43, p<0.001 |
|  |  |  | extension into the ACF |  | OR 1.92, 95% CI 1.03-3.6, p=0.038 |
|  |  |  | Age <40 (ref. ≥65 years) | Postoperative CSF leak | OR 5.3, 95% CI 1.17-24.11, p=0.030 |
|  |  |  | Age 40-64 (ref. ≥65 years) |  | OR 7.9, 95% CI 1.88-33.4, p=0.005 |
|  |  |  | Gender (ref. female*) |  | OR 2.4, 95% CI 1.24-4.63, p=0.010 |
|  |  |  | BMI ≥30 (ref. <30) |  | OR 2.10, 95% CI 1.14-3.86, p=0.017 |
|  |  |  | Previous radiation |  | = |
| **Supplementary table 2** Summary of study characteristics of prognostic cohort studies *(continued)* | | | | | |
|  |  |  | Intraventricular extension |  | OR 9.49, 95% CI 2.97-30.26 |
|  |  |  | Intraventricular extension | Intracranial infection | OR 11.91, CI 95% 3.64-38.95; p<0.001 |
|  |  |  | Previous radiation | Bleeding (ICA injury) | OR 44.00, 95% CI 3.73-519.00, p=0.003 |
|  |  |  | Intraventricular extension |  | OR 13.20, 95% CI 1.35-128.91, p=0.026 |
|  |  |  | extension into the ACF | Bleeding (intracranial bleeds) | OR 4.41 95% CI 2.04-9.51, p<0.001 |
|  |  |  | History of extrasellar tumor | Cranial nerve injury | OR 5.94, 95% CI 1.26-28.06, p=0.025 |
| Cavallo et al.,  2014 | High Risk | Consecutively treated craniopharyngiomas, N=83, adenoma 0% | Third ventricle involvement | Postoperative CSF leak | = |
| Cerina et al., 2016 | Low Risk | Consecutively treated newly diagnosed pituitary adenomas,  N=70 | Age (continuous) | Adrenal insufficiency | = |
|  |  |  | Gender (ref. male) |  | = |
|  |  |  | Tumor size 23 mm (ref. 17 mm) |  | OR 1.070, 95% CI 1.013-1.129, p=0.015^+^ (adjusted for tumor type) |
|  |  |  | Tumor type |  | = |
|  |  |  | Preoperative prolactin |  | = |
|  |  |  | Preoperative T4 |  | p=0.008 |
|  |  |  | Preoperative TSH |  | = |
|  |  |  | Preoperative IGF-1 |  | p=0.039 |

| **Supplementary table 2** Summary of study characteristics of prognostic cohort studies *(continued)* | | | | | |
| --- | --- | --- | --- | --- | --- |
|  |  |  | Preoperative Testosterone |  | = |
|  |  |  | Preoperative FSH |  | p=0.016 |
|  |  |  | Preoperative LH |  | p=0.001 |
|  |  |  | Preoperative cortisol |  | = |
|  |  |  | Preoperative urinary-free cortisol (nmol/24h) |  | p=0.041 |
| Chabot et al.,  2015 | High Risk | Large (>3 cm) or giant (>4 cm) pituitary macroadenomas, N=39 | Maximum tumor diameter | Complications in general | = |
|  |  |  | Knosp scores |  | = |
| Chohan et al., 2016 | High Risk | Large (>3 cm) or giant (>4 cm) pituitary adenomas, N=62 | Transverse length (>4 cm) | Permanent DI | p=0.02^+^ |
|  |  |  | Cranio-caudal length |  | = |
|  |  |  | Antero-posterior length |  | = |
|  |  |  | Maximum cross-sectional length |  | = |
|  |  |  | Tumor volume (>10 cm^3^) |  | = |
|  |  |  | Knosp 3-4 (ref. Knosp 1-2) |  | = |
| Chi et al.,  2013 | High Risk | Consecutively treated adenomas, N=80 | Learning curve  (cut off 40) | Postoperative CSF leak | = |
|  |  |  |  | Overall DI | = |
| Dallapiazza et al. 2014 | High Risk | Nonfunctioning macroadenomas with Knosp Grades 0–2, N=56 | Tumor volume | Postoperative CSF leak | = |
|  |  |  | Knosp grade (0 vs. 1 vs. 2) |  | = |
| **Supplementary table 2** Summary of study characteristics of prognostic cohort studies *(continued)* | | | | | |
| Dlouhy et al.,  2012 | Moderate Risk | Consecutively treated primarily sellar masses, N=92, adenoma 92% | Age (continuous) | Postoperative CSF leak | OR 0.93, 95% CI 0.88-0.98, p=0.008^+^ |
|  |  |  | Gender (ref. male) |  | = |
|  |  |  | BMI (per 5 kg/m^2^) |  | OR 1.61, 95% CI 1.10-2.29, p=0.016^+^ |
|  |  |  | Tumor volume (continuous) |  | = |
|  |  |  | Cushing's disease |  | = |
|  |  |  | Previous surgery |  | = |
| Gondim et al.,  2011 | High Risk | Consecutively treated pituitary adenomas, N=301 | supra-/parasellar extension | Postoperative CSF leak | OR 8.08; p=0.021 |
| Gondim et al.,  2015 | High Risk | Consecutively treated elderly (age ≥ 70 years) NFA patients vs. younger patients, N=374 | Age ≥70* (ref. <60 years) | Complications in general | p<0.05 |
| Hofstetter et al., 2012 | High Risk | Consecutively treated pituitary macroadenomas, N=71 | Tumor volume >10 cm^3^ | Complications in general | OR 6.3, 95% CI 1.6-25.0; p=0.008 |
|  |  |  | Tumor diameter >3 cm |  | OR 4.8, 95% CI 1.2-18.6; p=0.023 |
| Jakimovski et al., 2014 | High Risk | Consecutively treated pituitary adenomas, N=203 | Tumor size ≥2 cm (ref. <2 cm) | Postoperative CSF leak | = |
|  |  |  | Tumor volume  (per quartile) |  | = |
|  |  |  | Tumor type, functioning adenoma (ref. NFA) |  | = |
|  |  |  | Learning curve* (cut off 50) |  | p=0.004 |
| **Supplementary table 2** Summary of study characteristics of prognostic cohort studies *(continued)* | | | | | |
| Jang et al., 2016 | High Risk | Consecutively treated pituitary adenomas, N=331 | Age ≥50 years (ref. <50) | Complications in general | OR 2.75 95% CI 1.18-4.32,  p=0.047^+^ |
|  |  |  | Gender |  | = |
|  |  |  | Tumor size, macro- (ref. microadenoma) |  | OR 3.98, 95% CI 2.16-5.79, p=0.003^+^ |
|  |  |  | Knosp grade 3-4 (ref. 0-2) |  | OR 6.75, 95% CI 3.81-9.68,  p<0.001^+^ |
|  |  |  | Parasellar growth* |  | p=0.002 |
|  |  |  | Tumor type |  | = |
| Karnezis et al., 2016 | High Risk | Multicenter cohort study in pituitary adenomas and craniopharyngiomas, N=1161, 95% adenoma | Age (years) | Postoperative CSF leak | OR 0.982, 95% CI 0.967–0.997, p=0.022^+^ |
|  |  |  | Gender (ref. female*) |  | p=0.045 |
|  |  |  | BMI (kg/m2) |  | OR 1.033, 95% CI 1.007–1.059, p=0.014^+^ |
|  |  |  | ASA score |  | = |
|  |  |  | Charlson Index |  | = |
|  |  |  | Race |  | = |
|  |  |  | Recurrence |  | = |
|  |  |  | Prior skull-base operations |  | = |
|  |  |  | Radiotherapy* |  | p=0.007 |
|  |  |  | Chemotherapy |  | = |
|  |  |  | Myocardial Infarction |  | = |
|  |  |  | Chronic Heart Failure |  | = |
|  |  |  | Peripheral Vascular Disease |  | = |

| **Supplementary table 2** Summary of study characteristics of prognostic cohort studies *(continued)* | | | | | |
| --- | --- | --- | --- | --- | --- |
|  |  |  | Coronary Vessel Disease |  | = |
|  |  |  | Dementia |  | = |
|  |  |  | Chronic Pulmonary Disease |  | = |
|  |  |  | Connective Tissue Disease |  | = |
|  |  |  | Peptic Ulcer Disease* |  | p=0.029 |
|  |  |  | Mild Liver Disease |  | = |
|  |  |  | Diabetes Mellitus |  | = |
|  |  |  | Diabetes with Chronic Complications |  | = |
|  |  |  | Hemiplegia |  | = |
|  |  |  | Renal Disease |  | = |
|  |  |  | Any tumor |  | = |
|  |  |  | Leukemia |  | = |
|  |  |  | Lymphoma |  | = |
|  |  |  | Moderate or Severe Liver Disease |  | = |
|  |  |  | Metastatic Solid Tumor |  | = |
|  |  |  | Extension into ventricle |  | OR 3.585, 95% CI 1.693–7.592, p=0.001^+^ |
|  |  |  | Tumor type,  craniopharyngioma*  (ref: adenoma) |  | p<0.001 |
| **Supplementary table 2** Summary of study characteristics of prognostic cohort studies *(continued)* | | | | | |
| Leach et al., 2010 | High Risk | Consecutively treated pituitary fossa lesions, N=125, adenoma 87% | Learning curve (cut off 53) | Complications in general | = |
| Qureshi et al., 2016 | High Risk | Consecutively treated primary pituitary adenomas, N=78 | Learning curve (cut off 9) | Postoperative CSF leak | = |
|  |  |  |  | Transient DI | = |
|  |  |  |  | Permanent DI | = |
|  |  |  |  | Loss of pituitary function / panhypopituitarism | = |
|  |  |  |  | Sinusitis | = |
| Senior et al., 2008 | High Risk | Endoscopic transsphenoidal surgery, N=176, adenoma 84% | Age (continuous) | Postoperative CSF leak | = |
|  |  |  | Gender |  | = |
|  |  |  | BMI (continuous) |  | = |
|  |  |  | Tumor size >10* vs. <10mm |  | p=0.04 |
|  |  |  | Tumor size >20 vs. <20mm |  | = |
|  |  |  | RCC |  | OR 2.6; p<0.001 |
|  |  |  | Tumors other than adenomas |  | OR 9.0, p<0.001 |
|  |  |  | Gender | Overall DI | = |
|  |  |  | Tumor size (not defined) |  | = |
|  |  |  | RCC* |  | p=0.003 |

| **Supplementary table 2** Summary of study characteristics of prognostic cohort studies *(continued)* | | | | | |
| --- | --- | --- | --- | --- | --- |
|  |  |  | Tumor types other than RCC |  | = |
|  |  |  | Tumor location |  | = |
|  |  |  | Revision surgery |  | = |
| Sigounas et al.,  2008 | Moderate Risk | Endoscopic transsphenoidal pituitary surgery, N=105, adenoma 85% | Prior nonendoscopic surgery | Permanent DI | = |
|  |  |  | RCC* |  | p=0.028 |
|  |  |  | Tumor size (micro- vs. macroadenoma) |  | = |
|  |  |  | Prior endoscopic surgery |  | = |
|  |  |  | Gender (ref. male) | Overall DI | = |
|  |  |  | Race |  | = |
|  |  |  | Tumor size (micro- vs. macroadenoma) |  | = |
|  |  |  | NFA |  | = |
|  |  |  | Acromegaly |  | = |
|  |  |  | Cushing’s disease |  | = |
|  |  |  | Prolactinoma |  | = |
|  |  |  | RCC |  | 95% CI 2.0–25.8, p=0.003 |
|  |  |  | Craniopharyngioma |  | = |
|  |  |  | Prior endoscopic surgery |  | = |
| Thawani et al., 2017 | High Risk | Consecutively treated pituitary macroadenomas, N=203 | Age (continuous) | Postoperative CSF leak | = |
|  |  |  | Gender (ref. female) |  | = |
| **Supplementary table 2** Summary of study characteristics of prognostic cohort studies *(continued)* | | | | | |
|  |  |  | Tumor size |  | = |
|  |  |  | Cavernous sinus involvement |  | = |
|  |  |  | Prior surgery |  | = |
|  |  |  | Prior radiation |  | = |
|  |  |  | Age (continuous) | Infections (overall) | = |
|  |  |  | Gender (ref. female) |  | = |
|  |  |  | Tumor size |  | = |
|  |  |  | Tumor type, functional* (ref. non-functional) |  | p=0.04 |
|  |  |  | Prior surgery |  | = |
|  |  |  | Prior radiation |  | = |
|  |  |  | Age (continuous) | Overall DI |  |
|  |  |  | Gender (female) |  | = |
|  |  |  | Tumor size |  | = |
| Zhan et al., 2015 | High Risk | Elderly pituitary adenoma patients (≥65 years) compared to patients aged between 40 and 55 years, N=303 | Age ≥65 (ref. 40-55 years) | Postoperative CSF leak | = |
|  |  |  |  | Meningitis | = |
|  |  |  |  | Intracranial hematoma | = |
|  |  |  |  | Transient DI | = |
|  |  |  |  | Permanent DI | = |
|  |  |  |  | Overall DI | = |
|  |  |  |  | New hypopituitarism | = |
|  |  |  |  | Visual deterioration | = |

| **Supplementary table 2** Summary of study characteristics of prognostic cohort studies *(continued)* | | | | | |
| --- | --- | --- | --- | --- | --- |
| Zhang et al.,  2014 | High Risk | Transsphenoidal pituitary adenoma surgery, N=326 | Diabetes mellitus | Intracranial infections | OR 5.47, 95% CI 1,09-6,49; p=0,009 |
| * Prognostic factor has a higher chance of outcome  ^+^ Based on a multivariate analysis | | | | | |
